# AI Meets Attitudes: Cross-Sectional Quantitative Study of COVID-19 Vaccine Hesitancy in Alaska's Diverse Communities

**DOI:** 10.2196/81099

**Published:** 2026-07-07

**Authors:** Pritom Kumar Saha, Jinghui Guo, Latifur Khan, Renee F Robinson, Ubydul Haque

**Affiliations:** 1 Department of Computer Science, University of Texas at Dallas, Dallas, TX, United States; 2 See Acknowledgments; 3 Rutgers Global Health Institute, Rutgers University, 112 Patterson Street, New Brunswick, NJ, 08901, United States, 1 848-932-5827; 4 SFI MediaFutures – Research Centre for Responsible Media Technology & Innovation, Department of Information Science and Media Studies, University of Bergen, Bergen, Norway

**Keywords:** alaska public health, geographic disparities, trust in healthcare, culturally tailored interventions, public health

## Abstract

**Background:**

The global COVID-19 vaccine rollout faces challenges from persistent hesitancy, especially in rural and underserved regions. Alaska’s unique geographic, cultural, and infrastructural challenges create complex dynamics for vaccine uptake.

**Objective:**

This study uses machine learning on survey data to identify key sociodemographic and attitudinal predictors of hesitancy, informing targeted public health strategies.

**Methods:**

This study surveyed 720 Alaska adults, selected via targeted sampling to capture diverse COVID-19 vaccine attitudes across demographics and regions. A structured questionnaire assessed hesitancy through 17 indicators. We applied extreme gradient boosting, random forest, and K-nearest neighbors models for both regression and classification, and interpreted classification results via Shapley Additive Explanations values.

**Results:**

Analysis of 720 respondents showed that in Alaska, 1.8% (13/720) of surveyed individuals completed the full primary vaccination series (doses 1‐3) and received all 3 booster doses. A vaccination rate of 63.47% (at least 1 dose), with Pfizer preferred over Moderna. A total of 34% (238/720) of participants reported receiving the first dose of the COVID-19 vaccine, 43% (310/720) received the second dose, 18% (130/720) received a third dose, 22% (158/720) received the first booster, 13% (94/720) received the second booster, and only 4% (29/720) received a third booster. Geographic data revealed higher uptake in urban centers and variability in rural areas. Young adult males exhibited the highest hesitancy, while lesbian, gay, bisexual, and transgender individuals showed the lowest. Trust in the health care system was the strongest predictor, confirmed by machine learning analyses.

**Conclusions:**

Focusing on a geographically and demographically distinct US population, this study advances the scientific understanding of vaccine hesitancy while informing context-sensitive public health strategies. The findings offer actionable evidence to guide targeted communication, equitable outreach, and data-driven policy in Alaska and similarly underserved regions across the United States, underscoring the importance of culturally tailored, trust-centered interventions to promote vaccine uptake and health equity.

## Introduction

The global rollout of COVID-19 vaccines represents one of the most ambitious public health undertakings in modern history. Yet, despite broad vaccine availability in high-income countries like the United States, vaccine hesitancy continues to hinder efforts to achieve community-level immunity and prevent severe illness [1]. Persistent disparities in vaccine uptake exist across geographic, socioeconomic, and cultural lines [2,3]. Rural and underserved regions often fall behind urban centers, especially in access to resources and opportunities. These patterns reflect not only logistical challenges but also deeply rooted beliefs, institutional mistrust, and complex social determinants of health [4].

Alaska provides a distinctive case study for examining these dynamics. As the largest and one of the most sparsely populated US states, it faces substantial barriers to equitable vaccine distribution, including extreme weather, long travel distances between communities, and limited health care infrastructure in rural areas. Its population is highly diverse, comprising Alaska Native communities, urban residents, and transient workers, each with unique historical and cultural relationships to public health systems [5]. Alaska’s COVID-19 vaccine rollout, supported by centralized public health, federal logistics, and Tribal Health Organizations, enabled early adult eligibility in 2021 and rapid coverage in remote and Alaska Native communities. Despite these successes, uptake later plateaued, highlighting that availability alone cannot maintain vaccination. Understanding evolving attitudes, trust, and pandemic fatigue is crucial for addressing hesitancy across different phases.

While previous reports have noted lower vaccine uptake in certain areas of the state, little is known about the attitudinal and demographic factors that influence individual decisions around COVID-19 vaccination [6]. Although many studies have explored vaccine hesitancy at the national or subnational level [7-9], few have focused on Alaska [10], and even fewer have used predictive modeling to disentangle the complex drivers of vaccine behavior [11]. Most existing analyses rely on descriptive statistics or traditional regression frameworks, which may fail to capture nonlinear interactions or latent behavioral patterns. Recent advances in machine learning offer powerful tools to address these limitations, enabling researchers to identify high-risk subpopulations and uncover nuanced combinations of beliefs, demographics, and contextual factors that drive hesitancy [12,13]. However, the utility of such models relies heavily on their transparency. Recent systematic reviews underscore the necessity of interpretability for building trust and ensuring the usability of artificial intelligence (AI)–driven decision support systems. A meta-analysis of explainable AI methods in health care identified Shapley Additive Explanations (SHAP) as the most widely adopted approach for structured clinical data, owing to its capacity to provide both local and global feature attributions [14,15]. Unlike model-specific approaches or image-based techniques, such as Grad-CAM, SHAP offers a model-agnostic framework that is particularly well-suited for identifying complex behavioral drivers. This helps address concerns about the “black box” nature of many predictive models.

To address these challenges and fill knowledge gaps, we conducted a comprehensive survey of 720 residents across Alaska to investigate patterns of COVID-19 vaccine uptake, hesitancy, and future intent. Using both regression and classification approaches, we applied extreme gradient boosting (XGBoost), random forest, and K-nearest neighbors (KNN) models, along with dimensionality reduction (principal component analysis [PCA]) and model interpretation methods (SHAP). Our analysis aimed to (1) identify sociodemographic and attitudinal predictors of vaccine hesitancy, (2) evaluate how well these factors can predict future vaccination intent, and (3) generate regionally tailored insights to inform public health interventions.

## Methods

### Sampling Method and Participant Characteristics

This study recruited Alaska adults (≥18 y) using a multistage, geographically stratified sampling strategy to capture the state’s dispersed population and diverse settlement patterns. In stage 1, Alaska was stratified by boroughs and census areas, with census-derived “plots” representing coherent residential units classified as urban or rural. Stage 2 involved randomly selecting plots within each stratum, proportionally covering major urban centers (eg, Anchorage and Fairbanks) and rural regions to minimize urban bias. In stage 3, multiple individuals per plot (8-15) were recruited through online panels with the help of TGM Research [16] and Hays Research Group [17], local institutions, rural health facilities, social media, and community outreach (eg, flyers). This mixed-mode approach enhanced participation in remote areas. Overall, the framework ensured geographic representativeness across Alaska, capturing variation in urbanicity, health care access, and sociocultural context, which supported a robust analysis of vaccine hesitancy while substantially improving coverage compared with convenience or urban-focused sampling [18].

We used a targeted sampling approach to recruit individuals with diverse views on COVID-19 vaccination, including hesitant, undecided, and vaccinated participants. Data were collected between June and December 2024, during a later phase of Alaska’s COVID-19 vaccination campaign. By then, primary series were widely available, boosters had been introduced, and public messaging focused on long-term uptake. Responses reflect evolving attitudes, including hesitancy, booster fatigue, risk perception, and trust in health institutions. Participant characteristics were stratified by urban versus rural residence, racial and ethnic groups (Alaska Native or American Indian, White, Black, Hispanic or Latino, Asian, and multiracial), and socioeconomic status, such as income and education level. While the recruitment aimed for comprehensive demographic coverage, it is important to note that specific sociodemographic details were provided by a subset of the total of 720 respondents. Specifically, ethnicity information was provided by 112 respondents, education level by 113 respondents, and monthly income by 101 respondents. However, due to high data sparsity and potential skewness arising from these low response rates, these demographic variables were excluded from the machine learning feature set to ensure robust, generalizable predictions.

The sample reflected racial and ethnic diversity, including Alaska Native, White, Black, Hispanic, and Asian populations. Recruitment also aimed to capture variation in socioeconomic status, educational attainment, and cultural backgrounds to support a comprehensive analysis.

We administered a structured questionnaire modeled after previous, validated instruments for assessing vaccine hesitancy [19]. Recruitment efforts used diverse channels, including TGM Research, Hays Research Group LLC, and social media platforms such as Facebook (Meta), Twitter, and LinkedIn. Additional outreach was conducted through public and private institutions, Alaska’s state health department epidemiologists, leaflet distribution, and postings in grocery stores, restaurants, and other public locations across Alaska. Citizen scientists, university student bodies, and rural health facilities also facilitated participation, ensuring broad representation and engagement from communities across the region.

We have conducted a comprehensive study on vaccine hesitancy involving 720 residents of Alaska. The data were collected through a structured survey designed to capture a wide range of information relevant to vaccination decisions. This included essential sociodemographic factors (such as age, gender, income level, educational attainment, and ethnicity) and responses to 17 specific hesitancy-related questions. These questions probed a variety of underlying constructs, including political beliefs, trust in health care institutions and scientific information, perceived risks of COVID-19 and vaccines, and willingness to be vaccinated in the future.

### Data Analysis

Vaccine hesitancy was measured using 17 survey items on attitudes, beliefs, and trust, summarized as a composite score and a PCA-derived latent score. Future vaccination intent was captured via a single 5-point Likert item. These definitions ensure alignment between survey constructs and modeling targets in regression and classification analyses. To examine predictors of vaccine hesitancy, we applied 2 complementary machine learning methodologies—multivariate regression and multiclass classification. These models were fitted to estimate adjusted associations between vaccine hesitancy and several key predictors—age, gender, trust in the health care system, health care access, and medical history.

Age was categorized into 5 predefined groups (0‐17 y, 18‐29 y [young adults], 30‐44 y, 45‐59 y, and 60 y or older), consistent with previous research on vaccine hesitancy. Gender was categorized as men, women, or LGBTQ+. To assess differences in vaccine hesitancy across these demographic groups, we conducted 1-way ANOVAs for age, men, and women. Post hoc Tukey’s honestly significant difference tests were applied for pairwise comparisons between age groups and men and women identities. Statistical effect sizes were reported as partial eta squared (*η*²).

In the multivariate regression approach, we aimed to predict a continuous measure of vaccine hesitancy. The 17 questions related to hesitancy were treated as target indicators. We then trained a machine learning model to predict this hesitancy level using respondents’ sociodemographic and health care information. For this task, an XGBoost regressor was selected due to its high performance and interpretability compared to random forest and KNN. To operationalize the concept of hesitancy, we used 2 different measurement strategies. First, a composite hesitancy score was calculated for each respondent by averaging their scores across the 17 relevant questions, each of which was rated on a 5-point Likert scale. This method provides a straightforward and interpretable measure of overall hesitancy. Second, to capture the underlying latent structure of hesitancy, we applied PCA to the 17 hesitancy indicators. The first principal component, which explains the largest amount of variance in the data, was extracted to capture latent hesitancy, reduce dimensionality, and minimize noise, and was used as a single, consolidated measure of hesitancy. This technique helps to reduce dimensionality and noise in the data. To validate the composite hesitancy score, we assessed the internal consistency of the 17 indicators, yielding a strong Cronbach α of 0.8530, exceeding the standard accepted threshold of 0.70 and the robust threshold of 0.80. PCA showed that the first component accounted for 35.14% of the total variance, with consistently positive factor loadings. Furthermore, the PC1 score was highly correlated with the unweighted mean of the indicators (*r*=0.9532), confirming the robustness of the measure.

Pearson correlation analysis was subsequently used to interpret the relationships between the input features and the predicted hesitancy scores.

For the second approach, we framed the problem as a multiclass classification task. Here, we focused on a single, direct question from the survey: “I intend to get vaccinated against preventable diseases in the future.” The participant’s response to this question, captured on a 5-point Likert scale (“Agree,” “Disagree,” “Neutral,” “Strongly agree,” and “Strongly disagree“), served as the target variable. This method allows us to predict the future vaccination intent category. We implemented and compared 3 different classification algorithms, that is, XGBoost, random forest, and KNN. To interpret the model’s predictions and quantify the influence of each feature on the classification outcome, we calculated SHAP values.

Multivariate regression and multiclass classification offer complementary insights into vaccine hesitancy. Regression models treat hesitancy as a continuous latent trait based on attitudinal indicators, whereas classification models predict future vaccination intent as a categorical outcome. Together, they link underlying beliefs to observable behavioral intentions.

We applied XGBoost, random forest, and KNN models for both regression and classification tasks. These methods were selected due to their strong performance on structured tabular data, robustness with moderate sample sizes, and interpretability, which is essential for informing public health interventions. Although modern deep learning models and transfer learning-based classifiers can achieve high predictive accuracy, they were not considered here because they typically require larger datasets and often operate as “black boxes,” limiting transparency needed for actionable policy insights.

### Ethical Considerations

This study was conducted in accordance with institutional and national ethical standards for research involving human participants. Ethical approval was obtained from the Rutgers University Institutional Review Board (Protocol #Pro2023002010; approved December 4, 2023). Written informed consent was obtained from all participants before enrollment. To protect participant privacy and confidentiality, all data were deidentified before analysis and stored in secure, access-restricted systems. Participants received US $75 in compensation for their time and participation in the study.

## Results

### Vaccine Preference Insights

Among surveyed individuals in Alaska, 1.8% (13/720) received all primary series doses (doses 1, 2, and 3) along with all 3 booster doses (booster 1, 2, and 3). A total of 5.28% (38/720) received 5 doses in various combinations, including primary series plus 3 boosters or other permutations involving 3 boosters. Moreover, 4 doses, combinations of the primary series, and up to 2 boosters were reported by 5.14% (37/720) of respondents. Three doses were received by 8.19% (59/720), typically in combinations such as doses 1‐3 without boosters or 2 doses with 1 booster. A 2-dose coverage, including combinations such as the primary series only or a single dose with 1 booster, accounted for 10.69% (77/720). A single-dose uptake without additional doses or boosters was reported in 33.06% (238/720). Notably, 36.53% (263/720) of individuals had not received any COVID-19 vaccine dose or booster (Table 1). Among those who were vaccinated, Pfizer was the most received vaccine, followed by Moderna. This preference may reflect factors such as initial availability, distribution logistics, and public perception of efficacy during the primary vaccination rollout period [20].

**Table 1.** COVID-19 vaccination coverage among surveyed Alaska residents (N= 720) by number of doses received.

COVID-19 vaccination in Alaska	n (%)
Doses 1, 2, 3, and booster 1, 2, 3	8 (1.8)
Five doses were taken (eg, Doses 1, 2, and booster 1, 2, 3, or Doses 1, 2, 3, and booster 1, 2, or Doses 1, 2, 3, and booster 1 and 3)	38 (5.28)
Four doses were taken (eg, Doses 1, 2, and booster 1, 2, or Doses 1, 2, 3, and booster 1, or Doses 1, 2, 3, and booster 3)	37 (5.14)
Three doses were taken (eg, Doses 1, 2, 3, and no booster, or Doses 1, 2, and booster 1, or Doses 2, 3, and booster 3)	59 (8.19)
Two doses were taken (eg, Doses 1, 2, and no booster, or Doses 1 and booster 1, or Doses 3, and booster 3)	77 (10.69)
Only one dose was taken (eg, Doses 1, and no booster, or Doses 2 or booster 1)	238 (33.06)
Neither any dose nor any booster was taken	263 (36.53)

**Figure 1.**
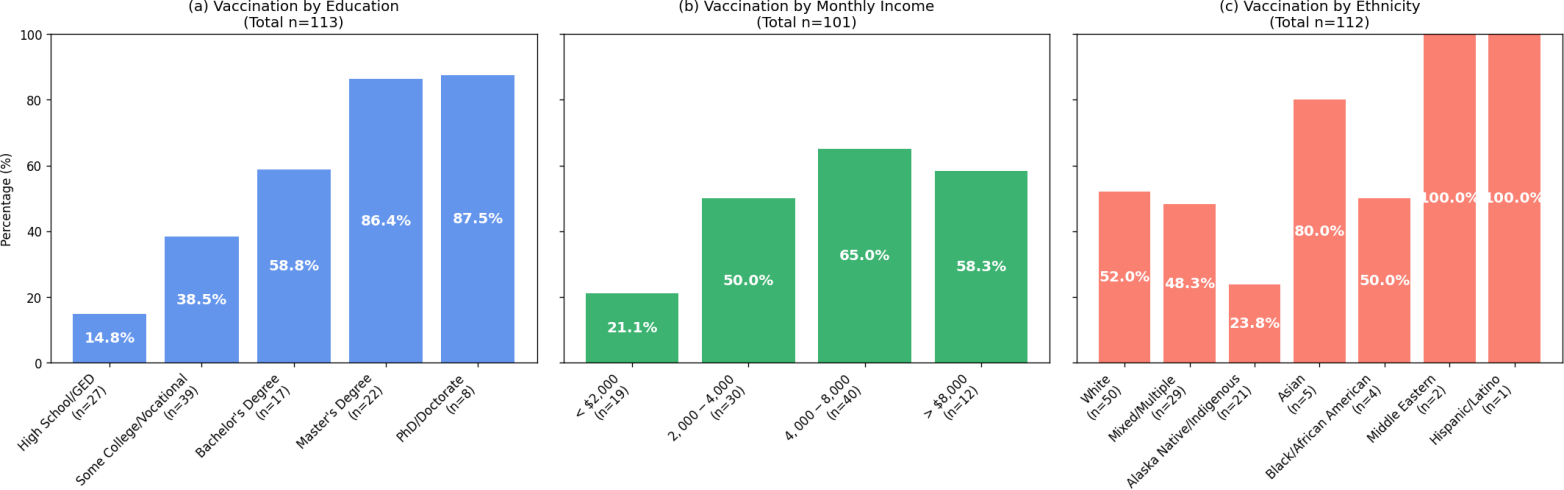
Vaccination uptake rates across sociodemographic subsets. The bar charts display the percentage of vaccinated respondents stratified by (A) education level, (B) monthly household income, and (C) racial or ethnic group. Percentages represent the proportion of vaccinated individuals within each specific category.

Analysis of the demographic subset provided further granularity regarding these uptake patterns (Figure 1). Vaccination rates demonstrated a strong positive association with educational attainment, rising sharply from 14.8% among respondents with a high school diploma or general educational development (n=27) to over 86% among those with a postgraduate degree (n=30). Economic factors similarly influenced behavior; respondents in the lowest income bracket (<US $2000 monthly) reported the lowest vaccination rate at 21.1%, whereas uptake peaked at 65% for those earning between US $4000 and US $8000. Disparities were also evident across racial and ethnic lines, specifically between the 2 largest respondent groups. A distinct gap was observed between White respondents (n=50), with a vaccination rate of 52%, and Alaska Native or Indigenous participants (n=21), whose coverage was notably lower at 23.8%. While data for other racial groups were collected, sample sizes were insufficient to identify reliable trends.

A detailed breakdown of the vaccine types administered, including the number of doses and the count of unique recipients, is presented in Table S1 in Multimedia Appendix 1. Figure S1 in Multimedia Appendix 1 illustrates the distribution of vaccine types.

### Vaccination Insights Based on Geography

Vaccinated respondents were widely distributed, with notable concentrations in major urban centers, such as Anchorage and Fairbanks, suggesting relatively higher vaccine uptake in these areas (Figure S2 in Multimedia Appendix 1). This geographic trend was quantified by stratifying the study population (N=720) into urban (n=418, 58.1%) and rural (n=302, 41.9%) residents. To ensure precise classification, urbanicity was determined by mapping respondent coordinates against the official qualifying urban area boundaries and radii defined by the 2020 Census [21]. As illustrated in Figure S3 in Multimedia Appendix 1, a direct comparison of vaccination rates within these subgroups reveals a notable disparity; 66.3% (277/418) of urban residents were vaccinated, compared with 59.6% (180/302) of rural residents. This 6.7% gap highlights that, while vaccine coverage was substantial across both settings, rural populations remained disproportionately unvaccinated (122/302, 40.4%) compared with their urban counterparts (141/418, 33.7%).

**Figure 2.**
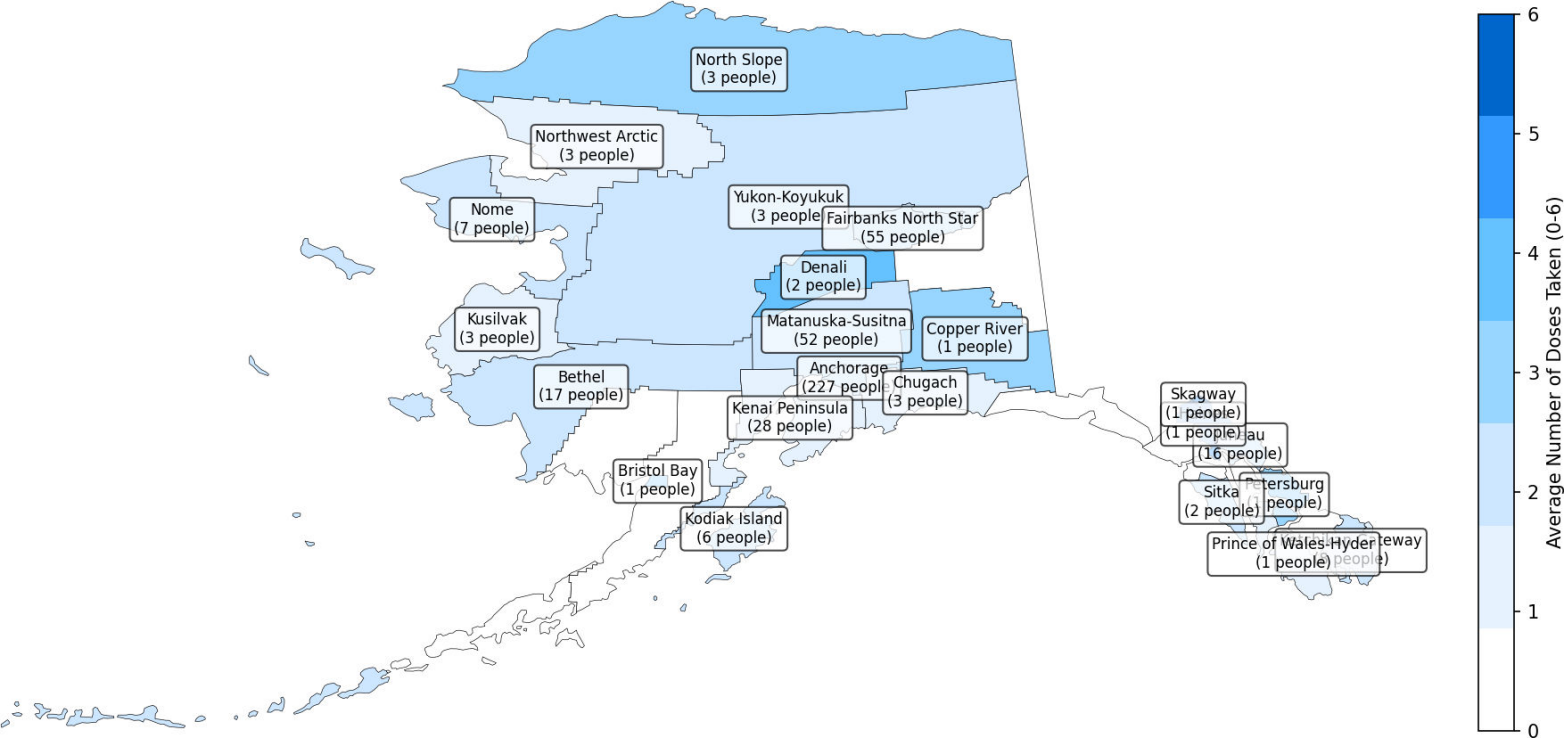
Geographic distribution of average level of COVID-19 vaccine dosage distribution in Alaska.

**Figure 3.**
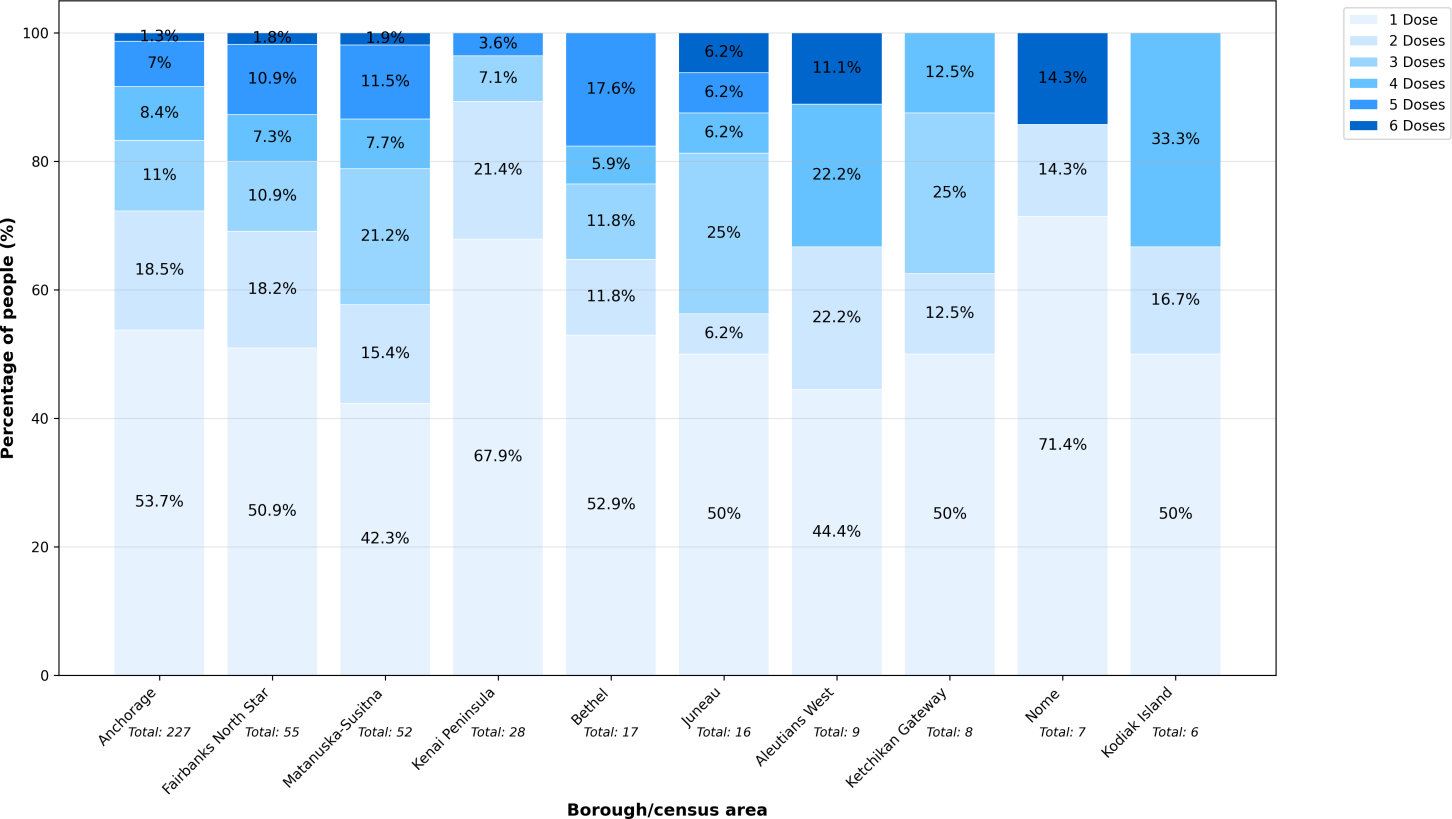
COVID-19 vaccination distribution by Alaska boroughs (top 10).

We further illustrated borough-level vaccination in Figure 2, which shows the average COVID-19 vaccination level across Alaska’s boroughs, ranging from 0 (unvaccinated) to 6 (fully vaccinated with booster 3). Each borough is shaded according to its vaccination rate, with darker shades of blue indicating higher rates. The figure shows notable variation in vaccination uptake; Denali, Haines, and Skagway exhibit the highest average vaccination level; however, the number of respondents was low in these areas. Several other regions, including Anchorage, Fairbanks North Star, and Matanuska-Susitna, exhibited moderate vaccination levels, with a high number of vaccinated individuals. Boroughs with fewer respondents generally exhibit lower or intermediate vaccination levels, highlighting disparities that may be driven by location-specific factors, such as accessibility, public health resources, or community attitudes toward vaccination. The complete breakdown of the dosage taken by respondents across the top 10 boroughs is illustrated in Figure 3.

### Multivariate Regression Results

For multivariate regression, we trained XGBoost, random forest, and KNN models using the 17 hesitancy indicators as the target variables. During training, we performed 2 levels of cross-validation—one for hyperparameter tuning and the other for model evaluation. For hyperparameter tuning, we performed a grid search with 3-fold cross-validation. For evaluation, we used 10-fold cross-validation. This systematic approach ensures that the selected model parameters achieve optimal performance not just on the training data but also on unseen validation data. Grid exhaustively searches through a manually specified subset of the hyperparameter space. For each model, a set of potential hyperparameter values is defined. For example, for XGBoost, we used grid search to tune key hyperparameters, including the learning rate, maximum tree depth, number of estimators, subsample ratio, and column subsample ratio (colsample_bytree). These parameters directly affect model complexity and learning capability. The cross-validation process then evaluated each hyperparameter combination by splitting the training dataset into multiple folds, training the model on a subset of the data, and validating it on the remaining part. This process was repeated across all folds, and the average performance metric, which is the micro *F*_1_-score, is used to select the best-performing parameters. Table 2 shows that the XGBoost model achieved the lowest root mean square error of 1.045 (SD 0.036), the lowest mean absolute error of 0.852 (SD 0.030), and the highest *R*^2^ score (mean 0.179, SD 0.037), which makes the XGBoost model the best model out of the 3 for the multivariate regression task.

**Table 2.** Comparison of model performance metrics for multivariate regression based on vaccine hesitancy-specific features.

Model	RMSE^a^ (↓^b^), mean (SD)	MAE^c^ (↓), mean (SD)	*R*^2^ score (↑^d^), mean (SD)
KNN^e^	1.129 (0.043)	0.893 (0.033)	0.062 (0.039)
Random forest	1.050 (0.031)	0.859 (0.028)	0.172 (0.029)
XGBoost^f^	1.045 (0.036)	0.852 (0.030)	0.179 (0.037)

a RMSE: root mean square error.

b Downward arrows denote lower is better.

c MAE: mean absolute error.

d Upward arrow denotes higher is better.

e KNN: K-nearest neighbor.

f XGBoost: extreme gradient boosting.

**Figure 4.**
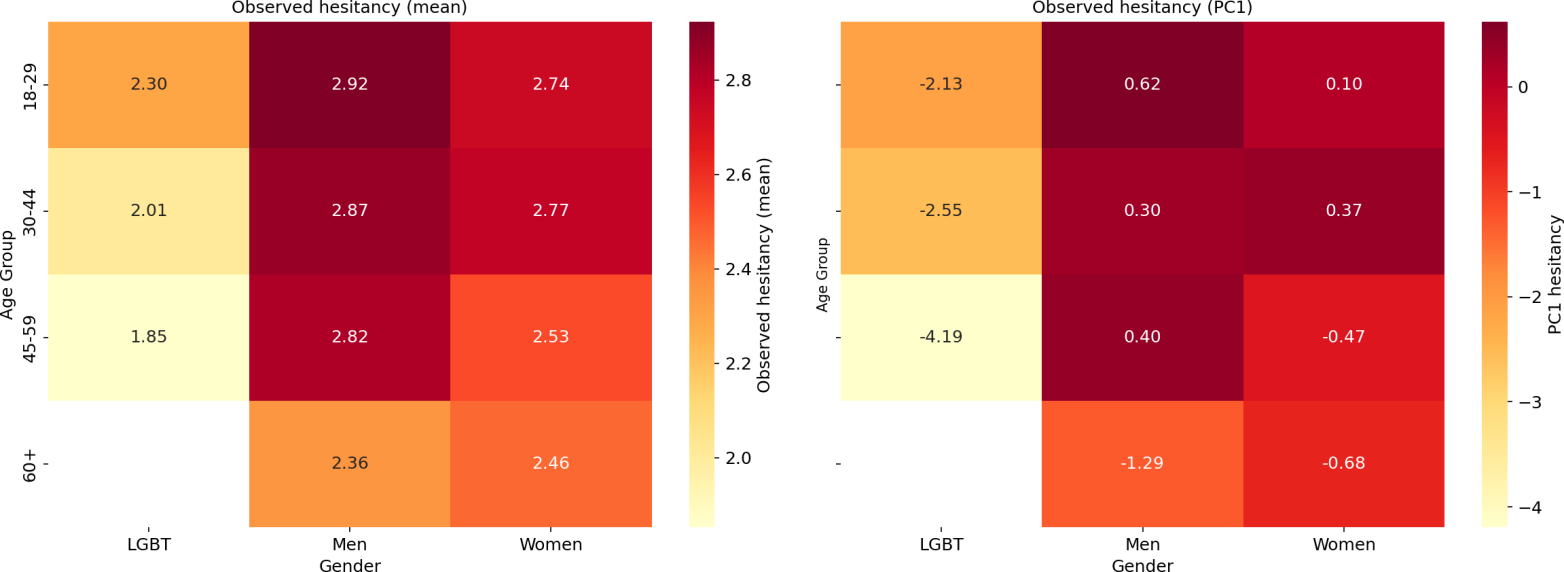
Observed hesitancy based on the mean score (left) and the PC1 score (right) across age, men, and women groups. Age groups were defined as 18‐29, 30‐44, 45‐59, and ≥60 years. LGBT: lesbian, gay, bisexual, and transgender.

The analysis of observed hesitancy, using both the average-score and PCA-based methods, yielded consistent demographic insights supported by significant ANOVA results. Men and women identity was a significant predictor of hesitancy (*F*=11.87, *P*<.001), with post hoc Tukey tests revealing a clear hierarchy; young adult males had the highest degree of vaccine hesitancy (Figure 4). More broadly, males in our sample demonstrated greater skepticism toward the COVID-19 vaccine compared with females (*P*=.01) and individuals identifying with the lesbian, gay, bisexual, and transgender (LGBT) community (*P*<.001). In contrast, individuals identifying with the LGBT community exhibited the lowest levels of hesitancy, suggesting a higher propensity for vaccine acceptance within this group. For age (*F*=10.93, *P*<.001), hesitancy decreased stepwise with increasing age, with significantly lower hesitancy in the 45‐59 group compared with ages 30‐44 years (*P*=.045), and further reductions in those aged ≥60 years compared with ages 45‐59 years (*P*=.04). Despite these clear patterns, age, men, and women were statistically significant predictors of vaccine hesitancy (*P*<.001), but the effect sizes for both age (*η*²=0.044) and gender (*η*²=0.032) remained small, suggesting that demographic factors alone do not account for much of the variance in vaccine acceptance.

**Figure 5.**
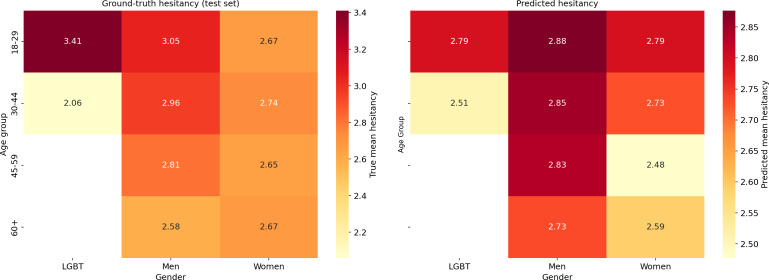
Observed (left) and predicted hesitancy (right) on the test set based on the mean score across age, men, and women groups. Age groups were defined as 18‐29, 30‐44, 45‐59, and ≥60 years. LGBT: lesbian, gay, bisexual, and transgender.

We then used the XGBoost regressor to predict the hesitancy scores derived from both method 1 (average score) and method 2 (PCA). The performance of these models on the held-out test set is visualized in Figures5  6. While the models successfully capture the general distribution of hesitancy scores, the predictions exhibit some variance from the true values. This suggests that while the models are learning relevant patterns, their predictive accuracy could be enhanced with a larger dataset and further hyperparameter tuning.

**Figure 6.**
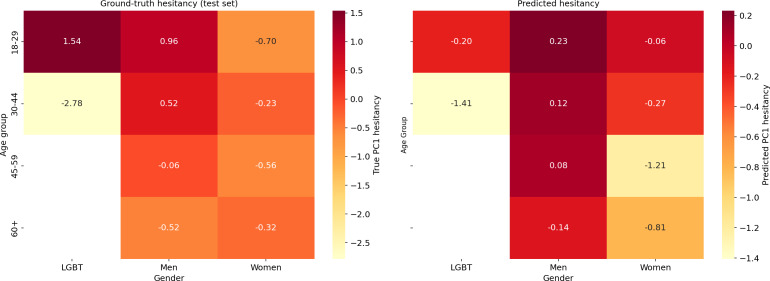
Observed (left) and predicted hesitancy (right) on the test set based on the PC1 score across age, men, and women groups. Age groups were defined as 18‐29, 30‐44, 45‐59, and ≥60 years. LGBT: lesbian, gay, bisexual, and transgender.

The trained XGBoost models also enabled examination of the relationships between input features and hesitancy. The Pearson correlation matrix revealed the strength and direction of linear relationships between variables (Figure S4 in Multimedia Appendix 1). Strong correlations were observed between hesitancy and factors related to personal beliefs and social influence.

**Figure 7.**
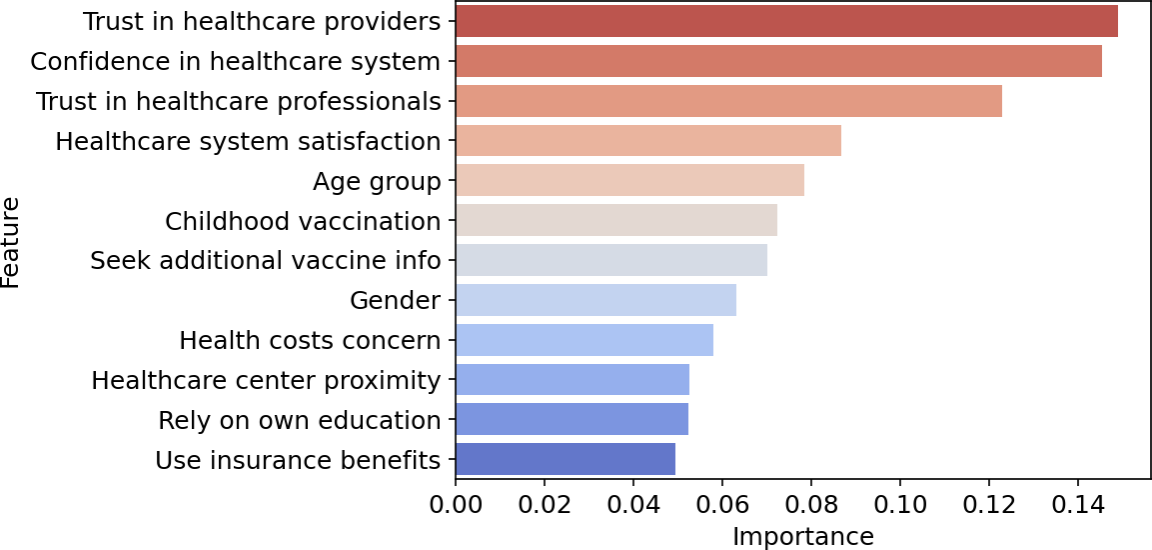
Feature importance influencing vaccine hesitancy.

Furthermore, a feature importance analysis was conducted using the trained XGBoost regressor to identify the most influential predictors of vaccine hesitancy. Trust in the health care system emerged as the most critical determinant across age, men, and women groups (Figures7  8), indicating that satisfaction with the health care system is the key feature influencing hesitancy across men and women groups.

**Figure 8.**
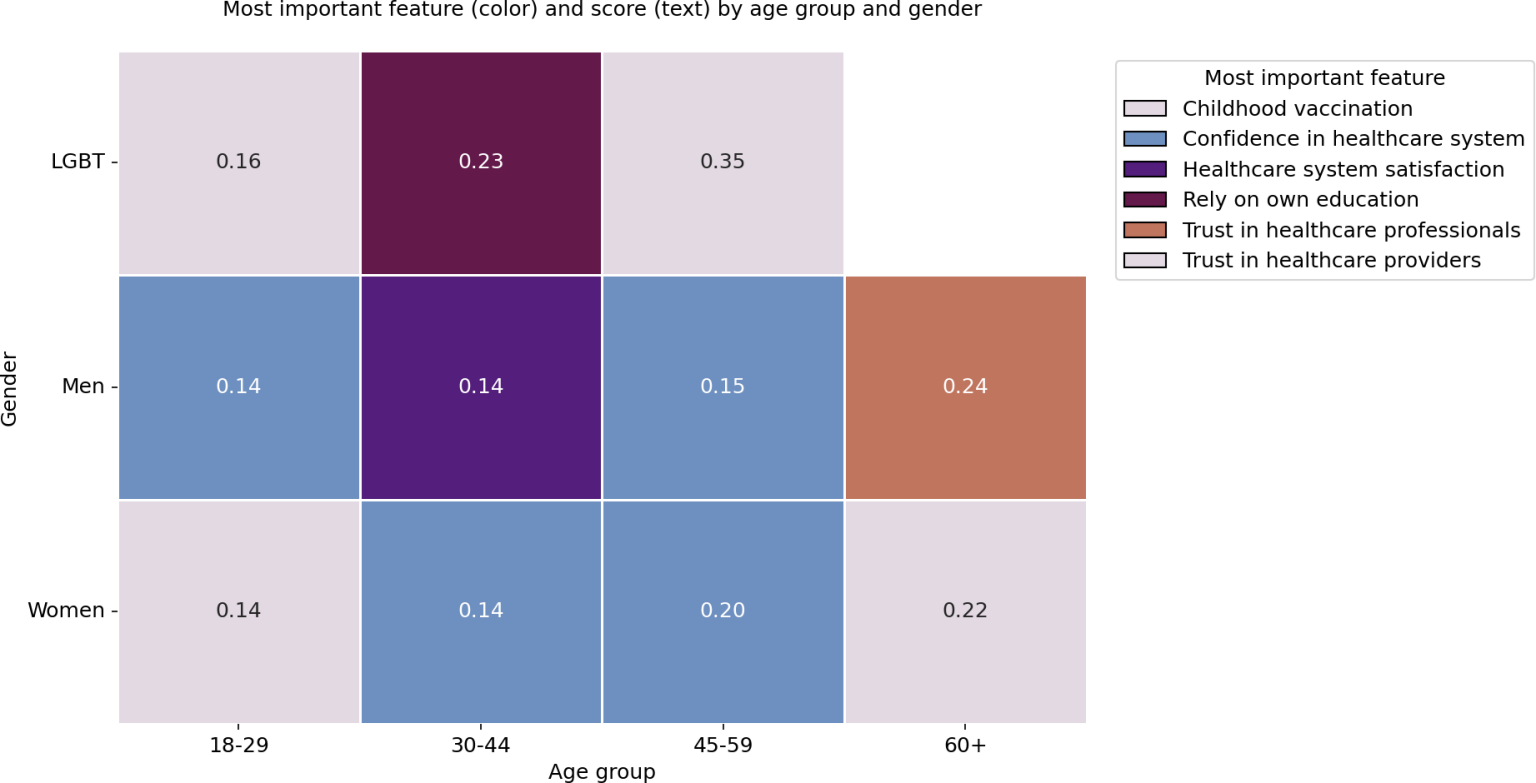
Feature importance influencing vaccine hesitancy for individual age, men, and women groups. LGBT: lesbian, gay, bisexual, and transgender.

### Multiclass Classification Results

In the multiclass classification task, we trained 3 machine learning models, XGBoost, random forest, and KNN, to predict future vaccination intent. To enhance model generalization and reduce the risk of overfitting, we applied grid search with cross-validation to tune hyperparameters for these 3 classifiers, as in the regression models.

A multiclass classification task aimed to predict hesitancy on a 5-point scale, using the question “I intend to get vaccinated against preventable diseases in the future” as the target. Here, the random forest model performed the best among the 3 models, achieving the highest micro and weighted *F*_1_-scores of 48.12% (SD 4.7%) and 44.19% (SD 5.08%), respectively, as presented in Table 3. We then performed SHAP analysis for each model. In SHAP plots, each point shows a feature’s contribution to an individual’s predicted hesitancy. Positive values increase hesitancy; negative values decrease it. Point color reflects the feature’s actual value, illustrating how high or low feature values influence predicted vaccine hesitancy. Figure S5 in (Multimedia Appendix 1) shows the average impact of each of the sociodemographic, trust in health care, and access to health care factors for each of the models. For all 3 models, confidence in the health care system and trust in health care providers were major determinants. For the KNN model, age group was also a major factor. These results are consistent with our regression results, which showed that trust and confidence in the health care system were also primary factors influencing vaccine hesitancy.

**Table 3.** Comparison of model performance metrics for multi-class classification based on vaccination willingness.

Model	*F*_1_ micro (↑^a^), mean (SD)	*F*_1_ weighted (↑), mean (SD)
KNN^b^	41.17% (5.47%)	38.66% (5.36%)
Random forest	48.12% (4.7%)	44.19% (5.08%)
XGBoost^c^	48.18% (5.39%)	43.76% (5.52%)

a Upward arrow denotes higher is better.

b KNN: K-nearest neighbors.

c XGBoost: extreme gradient boosting.

## Discussion

### Principal Findings

This study presents a comprehensive investigation of COVID-19 vaccine hesitancy in Alaska, highlighting key sociodemographic, geographic, and behavioral predictors by integrating survey data with machine learning techniques. Our results provide valuable insight into regional vaccination patterns, population-specific concerns, and predictors of future vaccination intent. These findings carry important implications for tailoring public health communication strategies and enhancing equity in vaccine uptake.

### Vaccination Patterns and Geographic Disparities

Our analysis revealed that 63.47% (457/720) of respondents had received at least 1 COVID-19 vaccine dose, with Pfizer being the most commonly administered, followed by Moderna. While this reflects moderate overall coverage, substantial geographic disparities were evident. Vaccine uptake was highest in urban centers such as Anchorage and Fairbanks, where health care access is stronger, and public health campaigns are more concentrated. In contrast, many rural and remote areas reported significantly lower vaccination rates, consistent with patterns observed in other frontier regions [22,23]. These disparities likely stem from a combination of structural barriers, such as limited access to health care services and logistical challenges, as well as sociocultural factors, including vaccine skepticism and lower trust in institutions. The clustering of unvaccinated individuals in specific communities highlights the influence of both access and attitudes [24]. To address these gaps, hyperlocalized interventions, such as mobile clinics, culturally tailored messaging, and engagement with trusted community leaders, are essential [25-27]. Such targeted efforts are critical to improving vaccine equity across Alaska’s diverse and geographically dispersed population.

### Demographic Correlations of Hesitancy

Our machine learning-based regression analysis identified young adult males as the demographic group most strongly associated with COVID-19 vaccine hesitancy, consistent with national and global trends. Importantly, the higher hesitancy observed among young adult males was supported by formal hypothesis testing and persisted after adjustment for socioeconomic and geographic factors, with effect sizes comparable with those reported in national and international studies of COVID-19 vaccine hesitancy. These findings also reflect persistent hesitancy and booster fatigue during the postinitial rollout phase, rather than early vaccine novelty or supply issues, highlighting Alaska’s uneven uptake despite initial vaccine availability. This hesitancy is often linked to lower perceived personal risk, skepticism, and limited engagement with health care systems [28,29]. In the US, vaccine hesitancy is driven by socioeconomic status, race, age, gender, education, insurance coverage, and political orientation [30,31]. In Alaska, the geographic dispersion of this group complicates outreach and access efforts. In contrast, individuals identifying as LGBT showed the lowest hesitancy, likely due to higher health literacy, stronger community engagement, and greater institutional trust, aligning with previous research on their proactive health behaviors [32]. These findings underscore the importance of tailoring public health messaging; peer-led campaigns, social media outreach, and culturally relevant storytelling may be effective for both hesitant and receptive groups. Age consistently emerged as the strongest predictor of vaccine intent [33,34]. Broader demographic drivers of hesitancy include socioeconomic status, race, education, gender, insurance coverage, and political affiliation [35]. Addressing these disparities through targeted, community-based strategies is crucial for enhancing vaccine uptake and fostering long-term public health resilience in Alaska.

### Trust in Institutions and the Role of Personal Beliefs

Consistent with frameworks such as the Health Belief Model, institutional trust emerged as the strongest predictor of vaccine hesitancy in both feature importance and correlation analyses [36]. This highlights the need for transparent, consistent, and culturally competent communication by trusted health authorities. In Alaska, this issue is magnified by a history of medical exploitation and systemic exclusion of Indigenous communities, which continues to erode trust in health care systems [37-39]. Addressing these deep-rooted challenges requires more than factual messaging; it demands culturally grounded strategies that honor tribal sovereignty, elevate Indigenous leadership, and foster long-term, respectful community partnerships. Nationally, individuals from distressed communities reported significantly lower trust in medical institutions (192/720, 26.6% vs 271/720, 37.6%) and vaccine confidence compared with those in more prosperous areas [40]. Similarly, in Alaska, political affiliation and median age were significant predictors of vaccine uptake, while media exposure had a limited influence [6]. Nearly two-thirds (461/720, 64%) of Alaskan interviewees reported concerns about vaccines, citing trust in information sources and personal experiences in their community as key factors in acceptance [41]. These findings underscore the complex interplay between trust, identity, and structural inequities in shaping vaccine attitudes across diverse populations. Addressing this requires culturally grounded strategies that honor tribal sovereignty, elevate Indigenous leadership, and build lasting partnerships based on respect, transparency, and community empowerment.

### Model Interpretability and Methodological Strengths

The integration of interpretable machine learning models, such as XGBoost and SHAP analysis, enhanced the transparency and policy relevance of our findings by identifying key predictors of vaccine hesitancy. Although the XGBoost regression and multiclass classification models showed moderate predictive performance (test accuracy of 48% in multiclass classification), they effectively captured important population-level behavioral patterns. The application of PCA to reduce the dimensionality of complex hesitancy measures further strengthened model robustness. However, our use of PCA was intended to assess whether a dominant latent dimension captured shared variance across attitudinal indicators rather than to develop a new psychometric scale. Exploratory analyses supported a general hesitancy factor, and PCA-based results were consistent with mean-score estimates. Future studies should apply validated hesitancy scales and longitudinal designs to further strengthen construct validity. These results underscore both the challenges of predicting nuanced behavioral intent with sociodemographic data alone and the potential for improvement with richer feature sets, including media consumption, political orientation, and misinformation exposure [42,43].

### Implications for Public Health Policy and Communication

Our findings have several actionable implications for public health planning in Alaska and comparable settings. Vaccine outreach must be geographically and culturally adaptive, as urban-centered strategies may not effectively reach rural or Indigenous communities, where hesitancy is shaped by structural and historical factors [44]. Strengthening tribally led health systems, deploying mobile vaccination units, and investing in community health workers are critical steps to increase access. Equally important is trust-building through empathetic, culturally respectful messaging delivered by local, trusted voices. Generalized campaigns are likely insufficient; instead, microtargeted approaches tailored to specific subgroups, such as young males or individuals with low institutional trust, are essential [45]. Historical injustices continue to influence public skepticism toward government, health care, and science, necessitating transparency and long-term community engagement [46,47].

To operationalize these targeted interventions at scale, policymakers should leverage policy-driven digital health frameworks. Recent systematic evidence indicates that mobile health apps and telemedicine platforms can significantly improve health care accessibility and chronic disease management in underserved regions when supported by robust governance [48]. For Alaska’s remote frontier communities, integrating the predictive insights from this study into digital health ecosystems such as SMS-based risk communication or telehealth-enabled counseling could overcome logistical barriers while maintaining the “human-in-the-loop” trust required for vaccine promotion.

Furthermore, moving beyond static analysis to real-time surveillance requires embedding these findings into smart health care systems. As highlighted in recent capability-oriented reviews, the current operational backbone of medical AI relies on “Narrow AI” and “Limited Memory” systems that use historical patient data for precise risk prediction [49]. Our SHAP-based model exemplifies this interpretable approach, offering a validated “Narrow AI” tool that can be integrated into public health dashboards. By adopting such smart health care architectures, health departments can transition from reactive measures to proactive, data-driven surveillance, identifying shifts in hesitancy trends early and deploying resources where they are most needed.

### Limitations

Despite the strengths of this study, including a large, diverse sample and the application of cutting-edge machine learning tools, several limitations should be noted. First, the survey sample, while geographically representative, may be subject to response bias. Individuals with strong views on vaccination may have been more likely to participate, potentially skewing estimates of overall hesitancy. Second, a cross-sectional design limits our ability to draw causal inferences about the directionality of relationships between predictors and hesitancy. Third, the reliance on self-reported data introduces potential measurement error, particularly in sensitive domains such as political beliefs or trust in institutions. Finally, although the stratified, plot-based sampling strategy enhanced geographic coverage, some remote areas had small numbers of respondents, which may limit fine-scale inference for sparsely populated regions.

Alaska’s dispersed geography, significant Indigenous population, and rural-urban divides shape patterns of vaccine hesitancy. While our machine learning models identify robust predictors locally, caution is needed when generalizing to other US states. Findings are most relevant to frontier or rural settings, with demographic stratification enhancing model interpretability and transparency.

### Strengths of the Study

This study’s strengths include its large, demographically diverse sample, its focus on a geographically unique and underserved region, and its use of transparent machine learning methods that balance predictive accuracy with interpretability. By focusing on Alaska, the study offers rare and valuable insights into vaccine hesitancy dynamics in a frontier context.

### Conclusion

This study offers data-driven insights into vaccine hesitancy in Alaska, highlighting distinct geographic and demographic patterns and the critical influence of institutional trust and personal beliefs. Trust in the health care system emerged as a central driver, with younger males showing the highest resistance and LGBT individuals the lowest. These findings underscore the need for culturally grounded, trust-centered, and locally tailored public health interventions. As Alaska and similar underserved regions face evolving public health challenges, addressing hesitancy through adaptive, inclusive, and emotionally resonant approaches will be vital for achieving equitable health outcomes across all communities.

## Supplementary material

10.2196/81099 Multimedia Appendix 1 Supplementary Table and Figures.

## Abbreviations

AI: artificial intelligence
KNN: K-nearest neighbors
LGBT: lesbian, gay, bisexual, and transgender
PCA: Principal Component Analysis
SHAP: Shapley Additive Explanations
XGBoost: extreme gradient boosting
