# Supplementary material for: AI Meets Attitudes: Cross-Sectional Quantitative Study of COVID-19 Vaccine Hesitancy in Alaska's Diverse Communities
Source: J Med Internet Res. 2026 Jul 7;28:e81099. doi: 10.2196/81099 (PMC13340900; doi:10.2196/81099)

**Multimedia Appendix 1: Supplementary Tables and Figures**

**Table S1: Distribution of vaccine types based on doses administered**

| **Vaccine** | **Doses Taken** | **Unique Recipient** |
| --- | --- | --- |
| Pfizer | 460 | 237 |
| Moderna | 257 | 118 |
| Novavax | 142 | 47 |
| Johnson & Johnson | 60 | 45 |
| Mixed (Pfizer & Moderna) | 36 | 10 |

**Fig. S1A: Distribution of COVID-19 vaccine types received.**

**
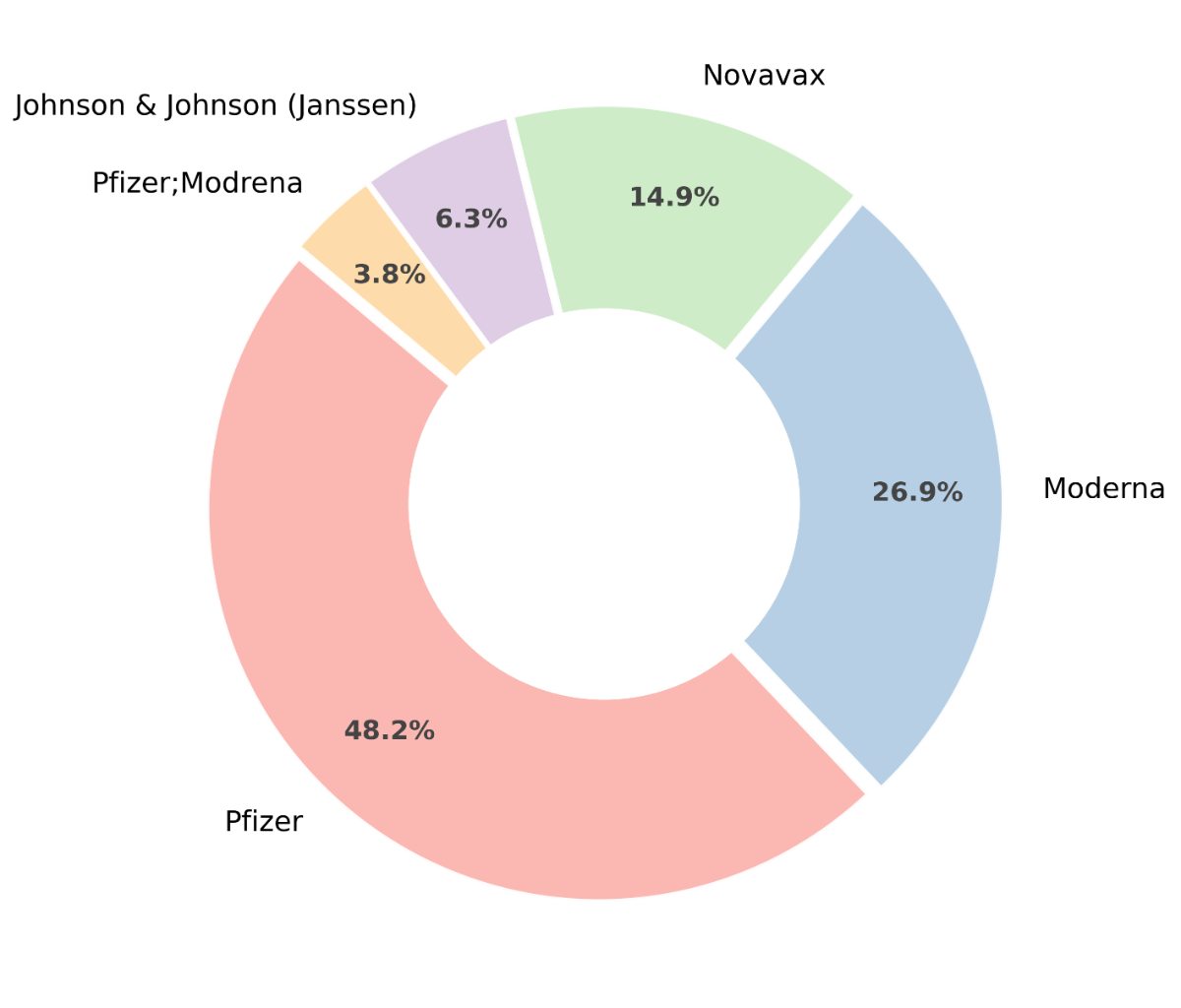
**

**Fig. S1B: Distribution of COVID-19 vaccine dosage received.**

**
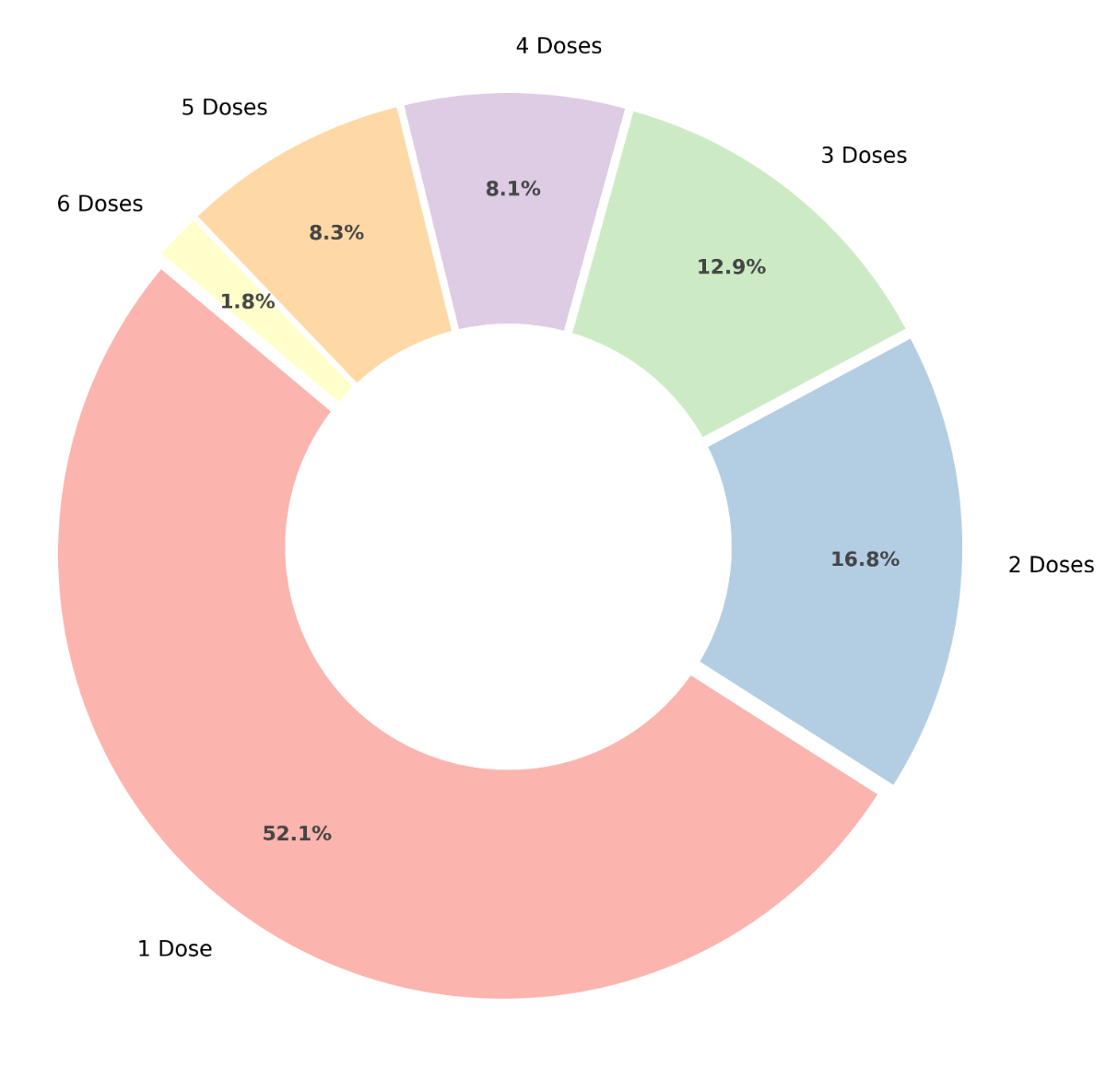
**

**Fig. S2: Geographic Distribution of COVID-19 Vaccination.**

**
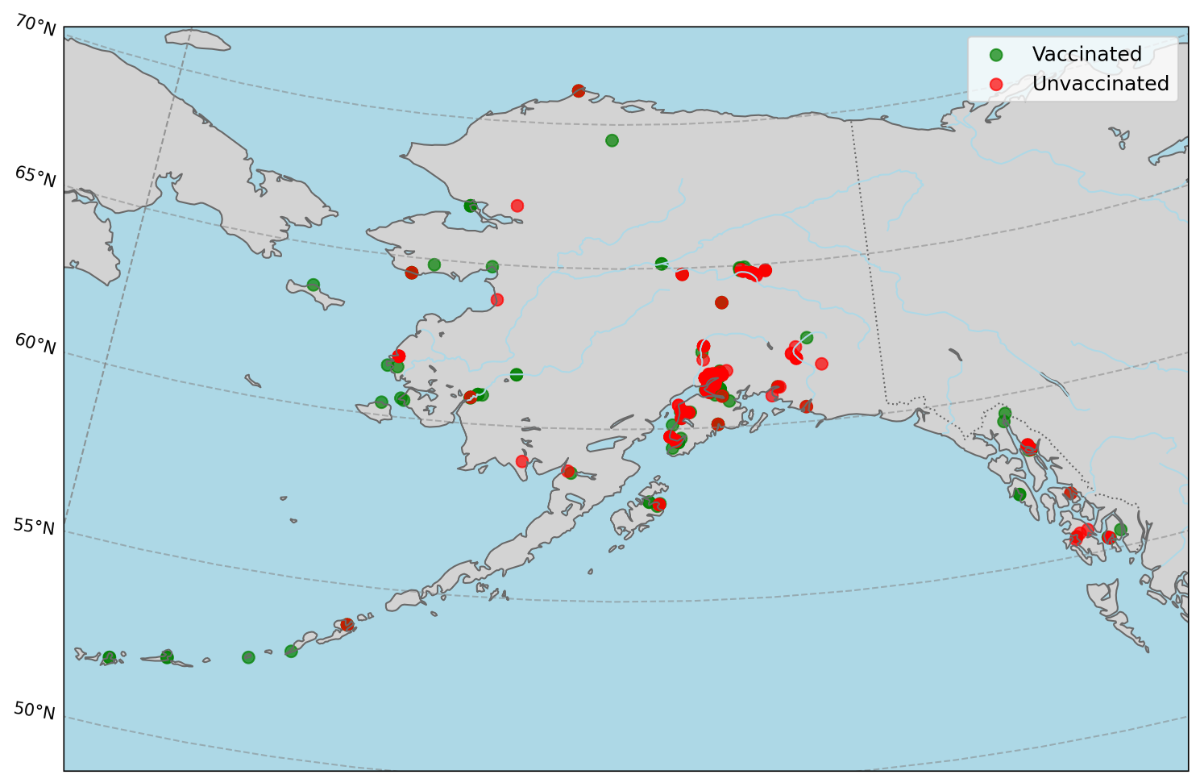
**

**Figure S3. Vaccination status stratified by urbanicity. The nested donut chart illustrates the distribution of the total study population (N=720) based on residential location and vaccination status. The inner ring divides respondents into Urban (58.1%, n=418) and Rural (41.9%, n=302) residents based on 2020 Census coordinate definitions. The outer ring further segments these groups into Vaccinated ("Vax") and Unvaccinated ("Unvax"). categories.**

**
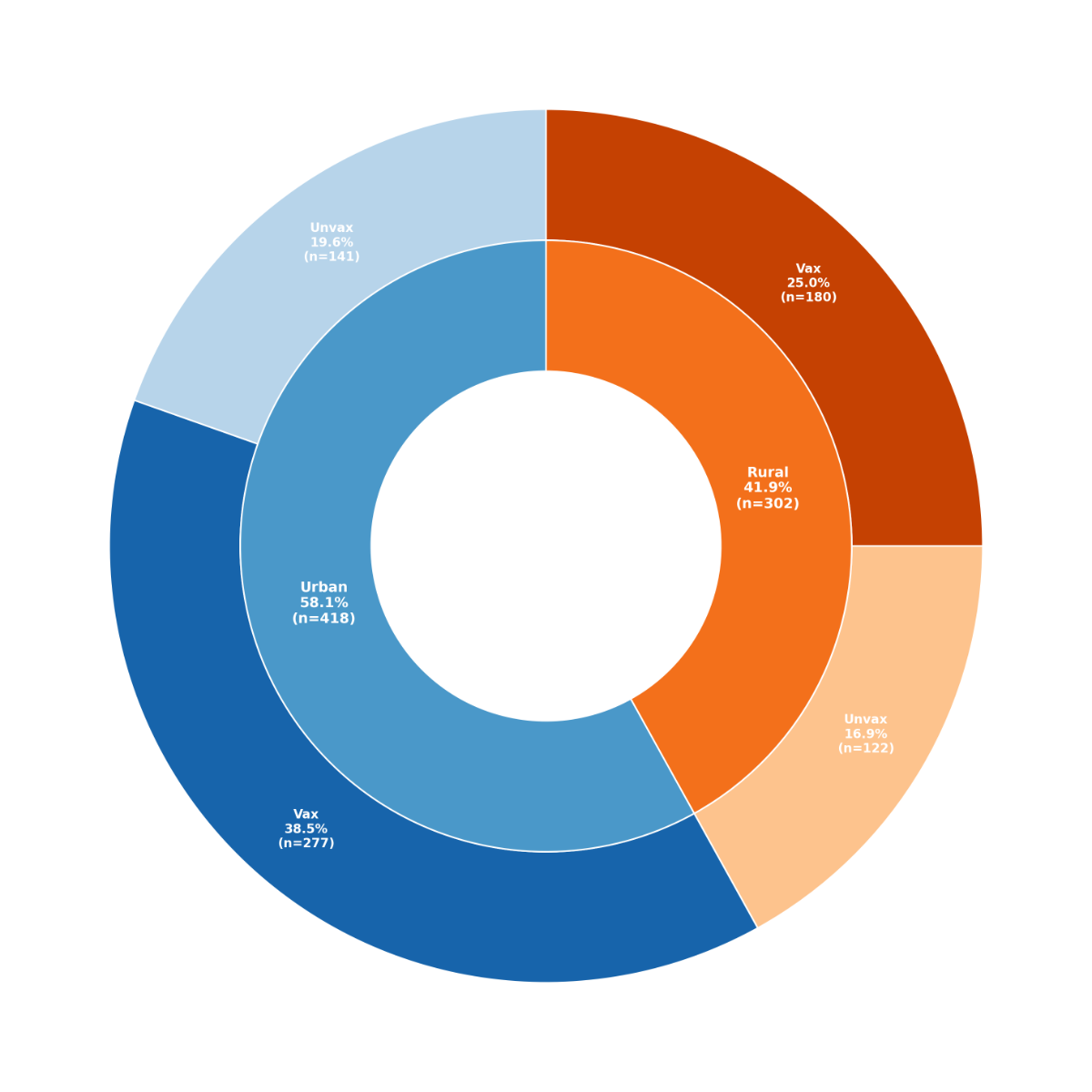
**

**Fig. S4: Pearson Correlation between hesitancy targets and age (left) & gender (right).**

**
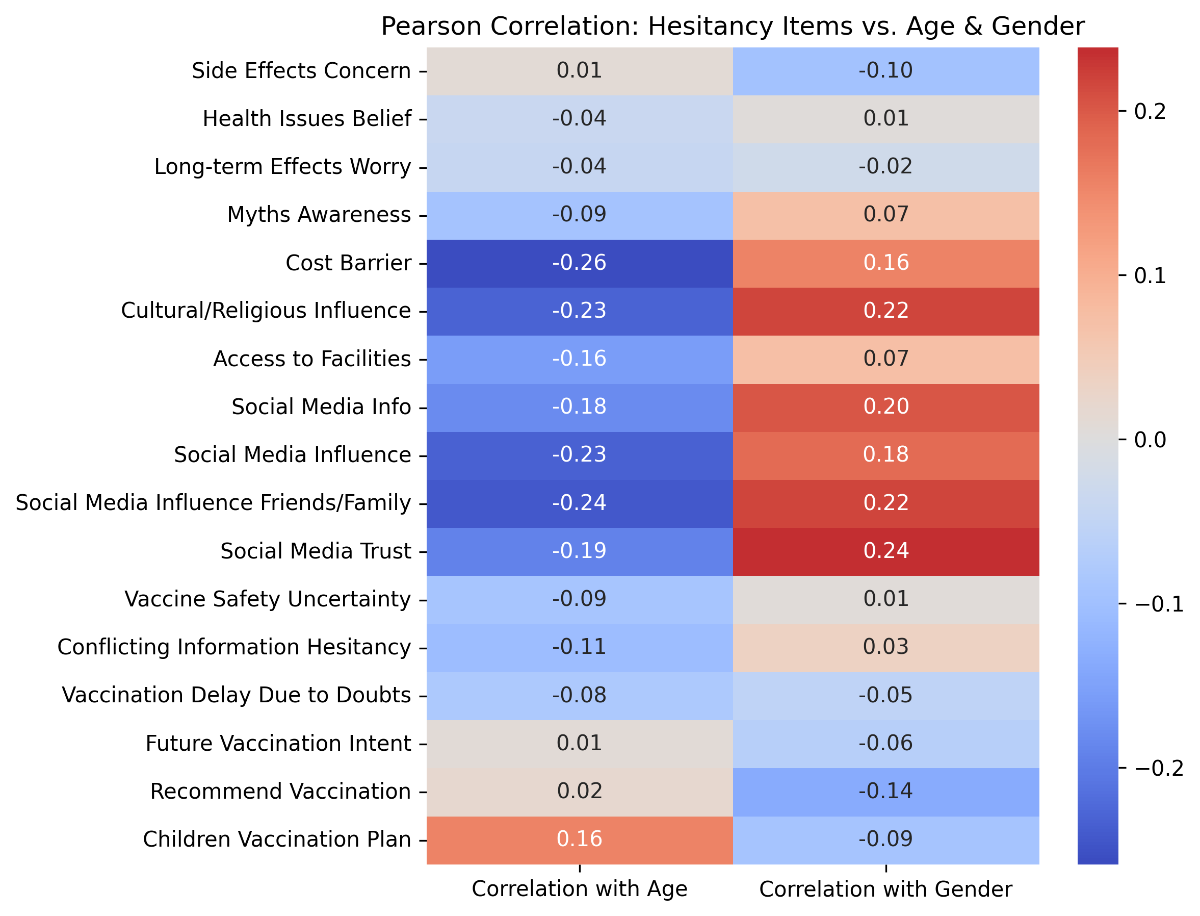
**

**Fig. S5A (KNN Classifier): Average impact on willingness to get vaccinated based on socio-demographic, trust, and availability of healthcare (mean ∥SHAP value∥).**

**
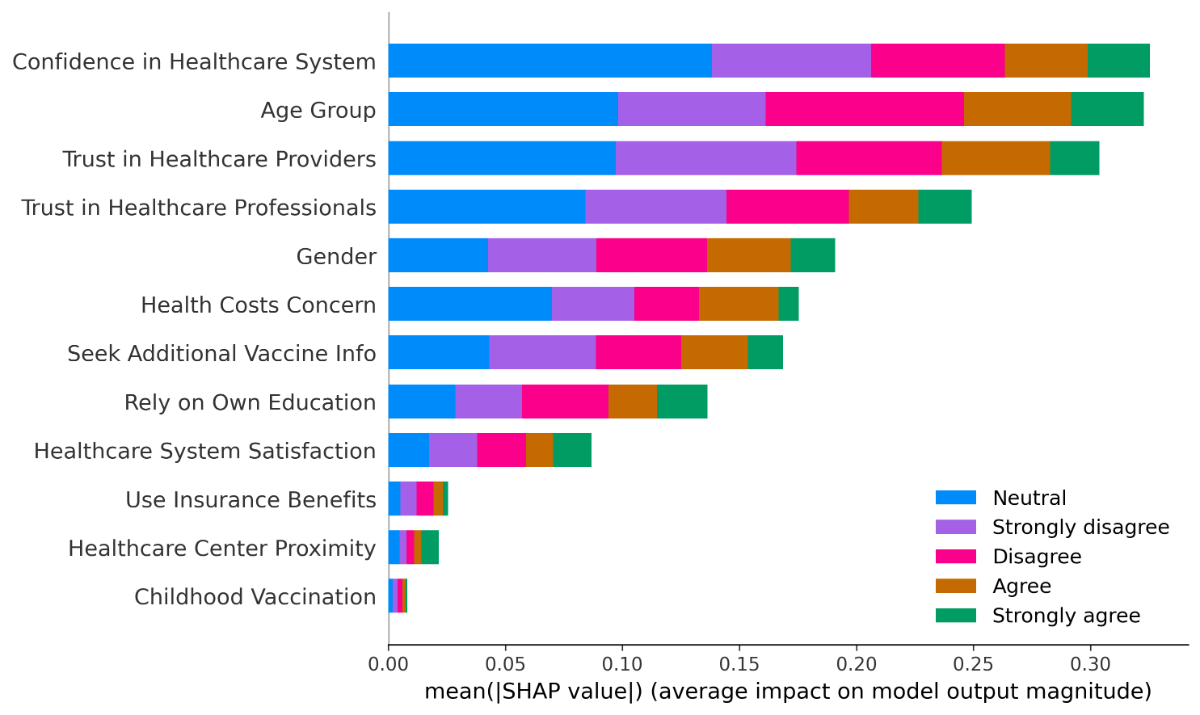
**

**Fig. S5B (Random Forest Classifier): Average impact on willingness to get vaccinated based on socio-demographic, trust, and availability of healthcare (mean ∥SHAP value∥).**

**
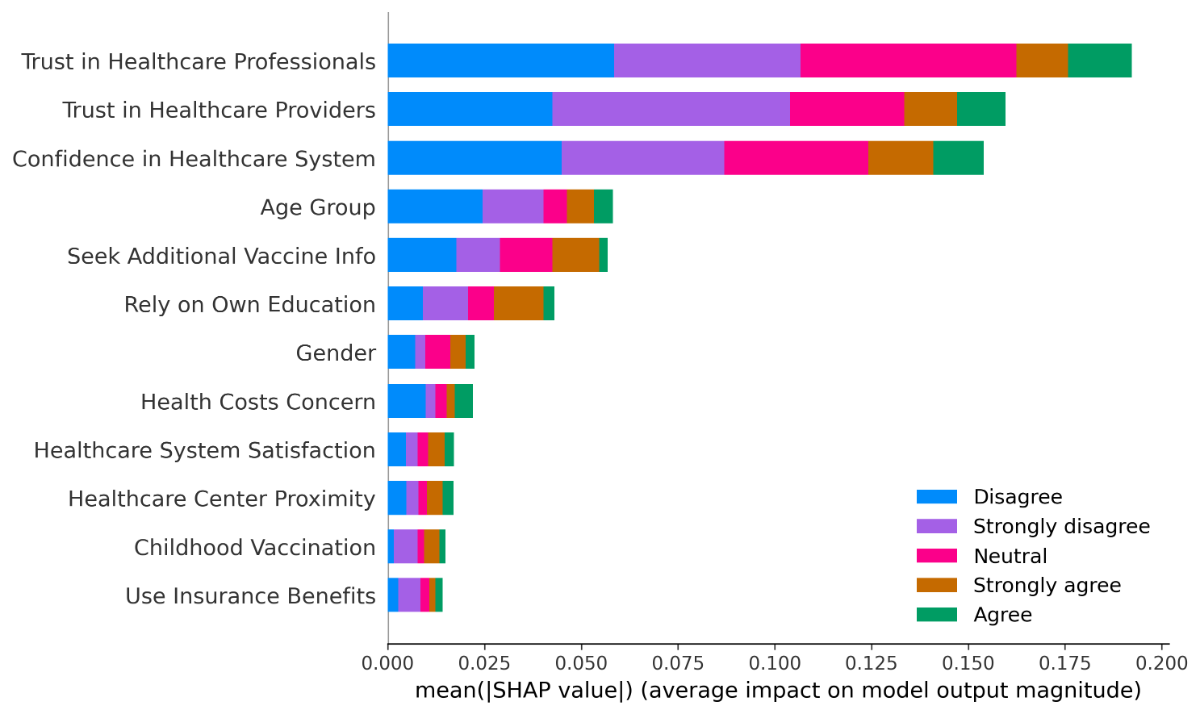
**

**Fig. S5C (XGBoost Classifier): Average impact on willingness to get vaccinated based on socio-demographic, trust, and availability of healthcare (mean ∥SHAP value∥).**


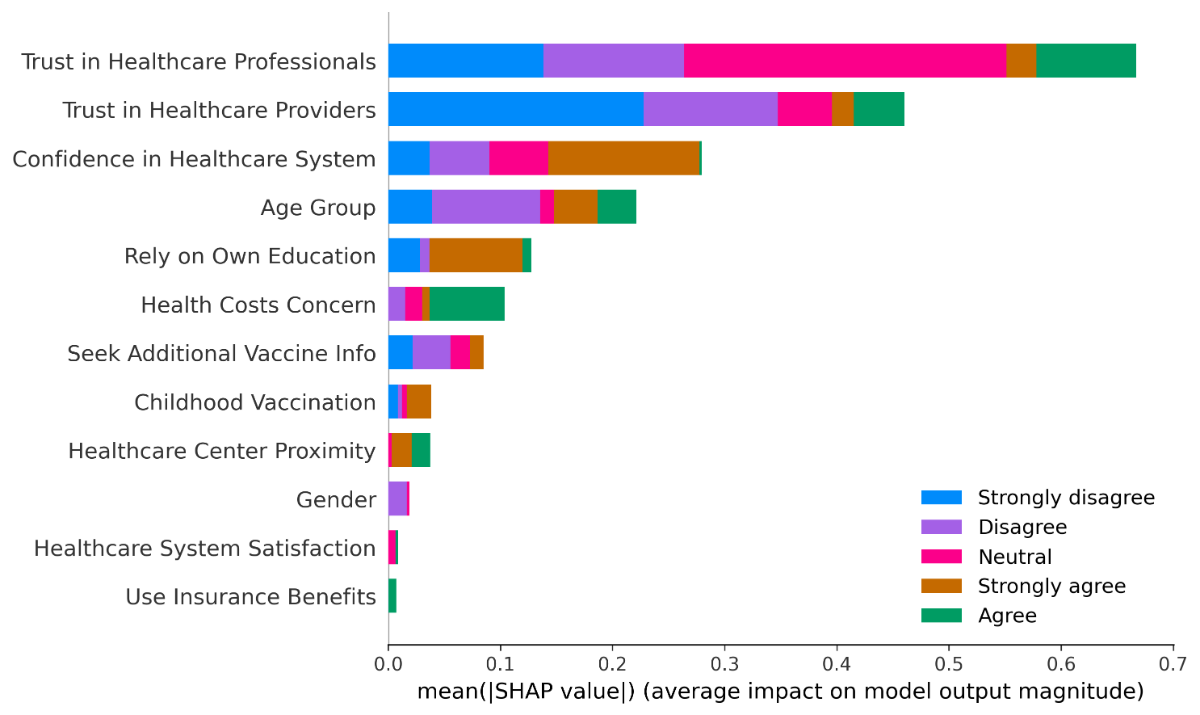

Supplement: Multimedia Appendix 1 [file jmir-v28-e81099-s001.docx]
